# Role of plaque inflammation in symptomatic carotid stenosis

**DOI:** 10.3389/fneur.2023.1086465

**Published:** 2023-01-24

**Authors:** Yilong Zheng, Mervyn Jun Rui Lim, Benjamin Yong-Qiang Tan, Bernard Poon Lap Chan, Prakash Paliwal, Ong Jia Yuan Jonathan, Chandra Bharatendu, Amanda Chee Yun Chan, Leonard Leong Litt Yeo, Joy Vijayan, Chiew S. Hong, Young Heng Chee, Lily Y. H. Wong, Jintao Chen, Victor Yao Feng Chong, Yanhong Dong, Chi Hsien Tan, Sibi Sunny, Hock Luen Teoh, Arvind Kumar Sinha, Vijay Kumar Sharma

**Affiliations:** ^1^Yong Loo Lin School of Medicine, National University of Singapore, Singapore, Singapore; ^2^Division of Neurosurgery, National University Health System, Singapore, Singapore; ^3^Division of Neurology, National University Health System, Singapore, Singapore; ^4^Gleneagles Medical Center, Singapore, Singapore; ^5^Alice Lee Centre for Nursing Studies, Singapore, Singapore; ^6^Department of Diagnostic Imaging, National University Health System, Singapore, Singapore

**Keywords:** endarterectomy, carotid stenting, patient selection, atherosclerosis, stroke

## Abstract

**Objective:**

Prior studies have shown that plaque inflammation on FDG-PET and the symptomatic carotid atheroma inflammation lumen-stenosis (SCAIL) score were associated with recurrent ischemic events, but the findings have thus far not been widely validated. Therefore, we aimed to validate the findings of prior studies.

**Methods:**

A single-center prospective cohort study that recruited patients with (1) recent TIA or ischemic stroke within the past 30 days, (2) ipsilateral carotid artery stenosis of ≥50%, and (3) were not considered for early carotid revascularization. The (1) maximum standardized uptake value (SUVmax) of the symptomatic carotid plaque, (2) the SCAIL score, and (3) stenosis severity of the symptomatic carotid artery were measured for all patients. The outcomes were (1) a 90-day ipsilateral ischemic stroke and (2) a 90-day ipsilateral symptomatic TIA or major adverse cardiovascular event (MACE).

**Results:**

Among the 131 patients included in the study, the commonest cardiovascular risk factor was hypertension (95 patients, 72.5%), followed by diabetes mellitus (77 patients, 58.8%) and being a current smoker (64 patients, 48.9%). The median (IQR) duration between the index cerebral ischemic event and recruitment to the study was 1 (0, 2.5) days. The median (IQR) duration between the index cerebral ischemic event and FDG-PET was 5 (4, 7) days. A total of 14 (10.7%) patients had a 90-day stroke, and 41 (31.3%) patients had a 90-day TIA or MACE. On comparison of the predictive performances of the SCAIL score and SUVmax, SUVmax was found to be superior to the SCAIL score for predicting both 90-day ipsilateral ischemic stroke (AUC: SCAIL = 0.79, SUVmax = 0.92; *p* < 0.001; 95% CI = 0.072, 0.229) and 90-day TIA or MACE (AUC: SCAIL = 0.76, SUVmax = 0.84; *p* = 0.009; 95% CI = 0.020, 0.143).

**Conclusion:**

Plaque inflammation as quantified on FDG-PET may serve as a reliable biomarker for risk stratification among patients with ECAD and recent TIA or ischemic stroke. Future studies should evaluate whether patients with significant plaque inflammation as quantified on FDG-PET benefit from carotid revascularization and/or anti-inflammatory therapy.

## Introduction

Atherosclerotic cervical carotid artery disease is a common cause of cerebral ischemia and is estimated to have affected 57.79 million people worldwide in 2020 (1). Carotid atherosclerosis is reported in 15%−20% of patients with a transient ischemic attack (TIA) or acute ischemic stroke in various population studies and hospital registries (2).

Routinely employed diagnostic imaging studies such as carotid duplex sonography, computed tomography angiography (CTA), and magnetic resonance angiography (MRA) provide reliable information about the plaque morphology and severity of stenosis. However, these modalities do not provide any information about the extent of plaque inflammation, an important cause of acute cerebral ischemia. Several methods have been proposed to non-invasively evaluate the extent of plaque inflammation, such as contrast-enhanced magnetic resonance imaging (3) and ^18^F-fluorodeoxyglucose positron emission tomography (FDG-PET) (4).

Previous studies have reported that plaque FDG uptake as evaluated shortly after the index cerebral ischemic event can predict both short- and long-term recurrent ischemic events among patients with significant carotid atherosclerosis (5, 6). The symptomatic carotid atheroma inflammation lumen-stenosis (SCAIL) score, a composite risk score comprising of stenosis severity and the degree of plaque inflammation, was also reported to be an independent predictor of both short- and long-term recurrent stroke (2, 6).

However, as the aforementioned studies were conducted mainly on Caucasian patients, the conclusions not be generalizable to patients of other ethnicities. Therefore, we aimed to evaluate the role of plaque inflammation (on FDG-PET), the SCAIL score, and the degree of stenosis in the prediction of 90-day recurrent ischemic events in our cohort of Asian patients with symptomatic carotid atherosclerosis.

## Methods

### Study population

This prospective single-center study included patients who presented with symptomatic carotid artery disease between 2016 and 2020. At our institution, all patients presenting with acute stroke or transient ischemic attack (TIA) are evaluated for carotid atherosclerosis with CTA and/or cervical duplex sonography. While patients with symptomatic carotid artery stenosis of ≥70% are considered for early revascularization, patients with 50%−69% stenosis are evaluated further for risk stratification.

Patients were included in the study if they (1) had a TIA or ischemic stroke within the previous 30 days, (2) were found to have ipsilateral carotid artery stenosis of 50% or more, and (3) were not considered for early carotid revascularization (carotid endarterectomy or carotid artery stenting within 14 days of the index event). Reasons for non-consideration for early carotid revascularization included a moderately severe stroke (NIHSS score of 12 or more points), concomitant acute ischemic heart disease, concomitant septicemia, and patients' refusal.

All study participants were evaluated clinically by credentialed neurologists, and all index and recurrent TIA or stroke events were confirmed with neuroimaging. Only patients with clearly defined focal motor, speech, or transient monocular vision loss were considered to have TIA. Patients with vague non-focal, sensory, or visual symptoms were excluded. Patients with an active malignancy, dementia, unstable cardiorespiratory disease, sepsis, prior neck irradiation, history of ipsilateral carotid surgery or stenting, who underwent carotid revascularization before FDG-PET imaging and/or had a competing stroke mechanism according to the TOAST classification (7) were also excluded.

All study participants received the standard of care treatment according to recommended guidelines (8), including antiplatelets, statins, cardiovascular risk factor control, and carotid revascularization where indicated.

This study was approved by the institutional ethics committee (Domain-Specific Review Board, National Healthcare Group, Reference Number 2015/00284), and written informed consent was obtained from all study participants and/or their legally-acceptable representatives.

### FDG-PET imaging

^18^F-FDG PET-CT was performed for all patients within 5 weeks of the index clinical event, using the Siemens Biograph mCT 64 slice CT. For patients who underwent carotid revascularization during the 90-day follow-up period, FDG-PET was done prior to carotid revascularization. For patients who did not undergo carotid revascularization within the 90-day follow-up period, FDG-PET was done within 5 weeks of the qualifying event.

All patients were requested to fast for a minimum of 6 h before intravenous ^18^F-FDG (320 MBq) was administered. PET imaging was performed 2 h later. PET images were acquired in a 3-dimensional mode in two-bed positions for 10 min each. A low-dose CT for attenuation correction was then done using the same scanner, followed by carotid CTA imaging from the aortic arch to the skull base using automated contrast-bolus tracking.

Following co-registration of the PET and CT images using a semi-automated algorithm with manual correction, carotid ^18^F-FDG activity corresponding to a 1 mm axial plaque slice was quantified by a study investigator (AKS) blinded to clinical outcomes.

### FDG uptake metric

^18^F-FDG uptake was quantified using standardized uptake values [SUV g/ml, defined as measured uptake (MBq/ml)/injected dose (MBq) per patient weight (g)]. The arterial segment with increased FDG uptake was defined relative to the slice of maximal stenosis on the co-registered CTA. We recorded mean and maximum SUV to quantify carotid plaque inflammation. Following semi-automated co-registration of PET and CT images, SUV was measured at 10 axial slices proximal and distal to the slice of maximal lumen stenosis and the adjacent internal jugular vein.

The Single Hottest Slice (SHS) was defined as the axial slice with the highest SUV uptake (SUVmax) (9). Intra-rater reliability assessment using Cronbach's alpha test revealed excellent internal consistency (α = 0.913, *p* < 0.001). No adverse events were reported by study participants after FDG-PET. FDG-PET imaging did not cause any delay in carotid revascularization in any patient. All recurrent cerebral ischemic events occurred after the FDG-PET scans.

### Plaque characteristics

The recorded plaque characteristics on cervical duplex imaging included size, presence of intraplaque hemorrhage, presence of plaque ulceration, and severity of stenosis.

Plaque size was measured with its length in the longitudinal view. A well-demarcated hypoechoic region within a carotid plaque represented intraplaque hemorrhage. An ulceration corresponded to an irregularity or break in the surface of the plaque and was considered significant if the recess was at least 2 mm deep and 2 mm long. The severity of carotid stenosis was graded as 50%−69% or 70%−99% according to the velocity criteria recommended by the Society of Radiologists in Ultrasound (10), and confirmed with CTA in all patients. All cervical duplex ultrasound studies were performed by credentialed sonographers (CSH, YHC) or a neurosonologist (VKS). The SCAIL score was derived according to the methodology described by Kelly et al. (2).

### Clinical and laboratory data collection

Clinical information collected includes age, sex, hypertension, diabetes mellitus, and history of smoking. High-sensitivity C-reactive protein (HS-CRP) levels in the blood were also measured for all participants on the day of recruitment. All participants were followed up for 90 ± 10 days after the index clinical event. Follow-up assessments were performed in person at 90 ± 10 days after the qualifying event.

### Exposures and outcomes

The exposures were stenosis severity, the SCAIL score, and the SUVmax of the symptomatic carotid plaque. The primary outcome was symptomatic ischemic stroke affecting the ipsilateral territory of the symptomatic carotid artery within 90 days of the index cerebral ischemic event. The secondary outcome was the occurrence of either a symptomatic TIA or a major adverse cardiovascular event (MACE, including stroke, acute myocardial infarction, and cardiovascular mortality) within 90 days of the index cerebral ischemic event.

For patients who underwent revascularization during the study period, strokes and TIAs that occurred within 90 days of the index event but after revascularization were not counted toward the study outcomes.

All neurological events were confirmed (1) clinically by a neurologist and (2) radiologically by CT/MR neuroimaging. All cardiac events were confirmed clinically by a cardiologist.

### Statistical analyses

The baseline characteristics of the study participants were reported using count numbers and percentages for categorical variables and mean and standard deviation for continuous variables. We used Pearson's χ^2^ test and student's *t*-test for categorical data and continuous variables for univariate analyses. Fisher's exact test was used for variables with a sample size of lesser than 5 under any category. A *p*-value of lesser than 0.05 was taken as statistically significant.

Multivariate time-to-event analysis adjusting for potential confounders was performed by constructing Cox proportional hazard models. Potential confounders adjusted for include age, sex, hypertension, diabetes mellitus, smoking history, plaque size, presence of plaque hemorrhage (11), presence of plaque ulceration (12, 13), and the HS-CRP level (14). Hypothesis testing was performed using the likelihood ratio test, with *p* < 0.05 considered statistically significant. Time-to-event was defined as the duration in days between the date of recruitment to the study and the date of occurrence of the first clinical outcome event within 90 days of recruitment to the study. Patients who underwent carotid revascularization during the study period were censored at the point of revascularization.

To determine whether the SCAIL score or SUVmax was superior in predicting 90-day events, hypothesis testing comparing their predictive performances on receiver operating characteristic (ROC) analysis was performed using DeLong's method. ROC analysis was not performed for the severity of carotid stenosis due to an insufficient number of categories (there were only two categories–50%−69% and 70%−99% stenosis).

**Figure 1:**
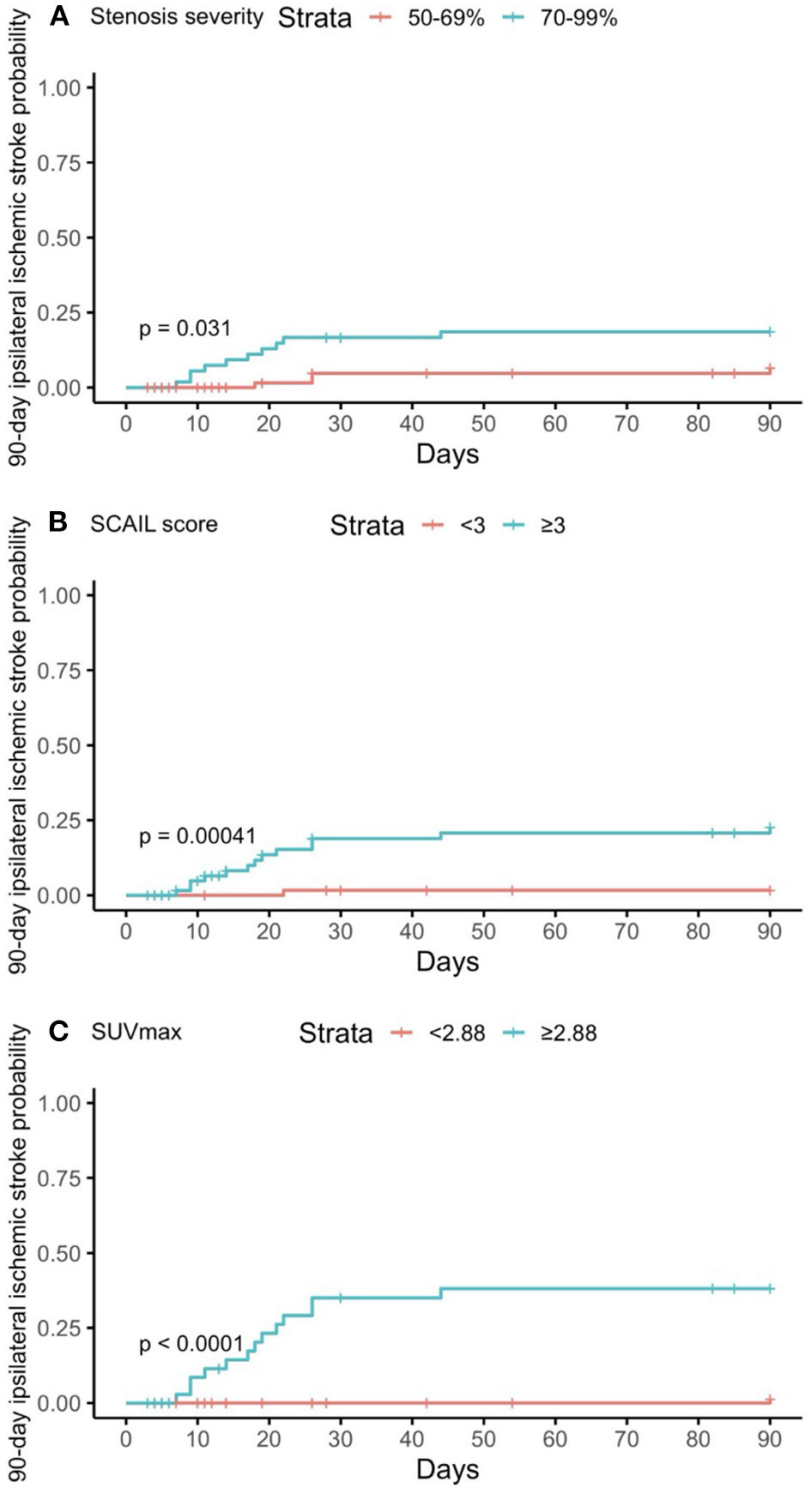
Kaplan–Meier curves of predictors for 90-day ipsilateral ischemic stroke. **(A)** Degree of stenosis in symptomatic plaque; **(B)** Symptomatic plaque SCAIL score; **(C)** Symptomatic plaque SUVmax. SCAIL, symptomatic carotid atheroma inflammation lumen-stenosis; SUVmax, maximum standardized uptake value.

**Figure 2:**
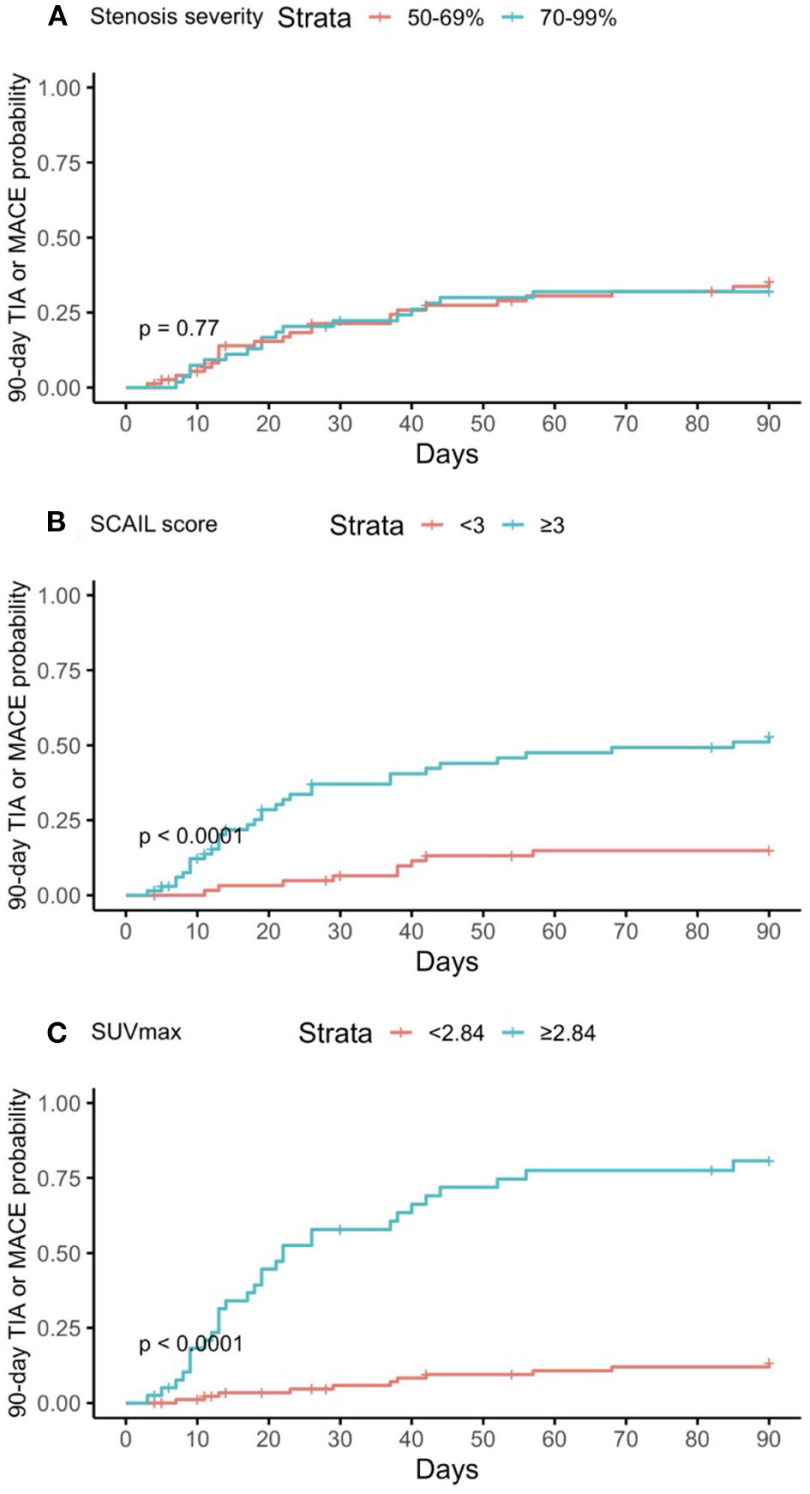
Kaplan–Meier curves of predictors for 90-day TIA or MACE. **(A)** Degree of stenosis in symptomatic plaque; **(B)** Symptomatic plaque SCAIL score; **(C)** Symptomatic plaque SUVmax. TIA, transient ischemic attack; MACE, major adverse cardiovascular event; SCAIL, symptomatic carotid atheroma inflammation lumen-stenosis; SUVmax, maximum standardized uptake value.

The threshold SCAIL scores and SUVmax values that gave the optimal balance of sensitivity and specificity for the prediction of 90-day events were also derived. The cohort was stratified according to the respective threshold values, and Kaplan–Meier plots were then constructed accordingly to illustrate the association between the exposures and 90-day events (Figures 1, 2). Patients who underwent carotid revascularization during the study period were censored at the point of revascularization.

All data analyses were conducted using R Studio Version 1.2.5042.

## Results

### Baseline characteristics of the study population

The baseline characteristics of the study population were reported in Table 1. During the study period, a total of 3,178 patients with TIA or acute ischemic stroke were admitted to our tertiary center, of which 239 (7.5%) were noted to have carotid stenosis of 50% or more. While 71 (29.7%) of the 239 patients underwent early carotid revascularization, 16 (6.7%) had a history of prior neck radiation, 2 (0.8%) had prior carotid stenting, and 19 (7.9%) refused to participate (Supplementary Figure 1). A total of 131 patients were included in the analysis.

**Table 1:** Baseline characteristics of the study population stratified by the degree of stenosis in the symptomatic carotid artery.

**Variable**	**50% −69% stenosis (*n* = 54, 41.2%)**	**70% −99% stenosis (*n* = 77, 58.8%)**	**Total (*n* = 131)**	***p*-Value**
Age; mean (SD)	64.4 (12.1)	65.3 (11.2)	64.9 (11.5)	0.653
Male; *n* (%)	46 (85.2)	65 (84.4)	111 (84.7)	1.000
**Ethnicity;** ***n*** **(%)**				
Chinese	40 (74.1)	51 (66.2)	91 (69.5)	0.069
Malay	9 (16.7)	8 (10.4)	17 (13.0)	
Indian	5 (9.3)	11 (14.3)	16 (12.2)	
Others	0 (0.0)	7 (9.1)	7 (5.3)	
Hypertension; *n* (%)	40 (74.1)	55 (71.4)	95 (72.5)	0.893
Diabetes mellitus; *n* (%)	20 (37.0)	34 (44.2)	77 (58.8)	0.526
Current smoker; *n* (%)	23 (42.6)	41 (53.2)	64 (48.9)	0.306
High-sensitivity C-reactive protein (mg/L); mean (SD)	8.4 (12.7)	13.4 (35.5)	11.4 (28.4)	0.325
**Characteristics of the symptomatic plaque at baseline**				
Size (mm); mean (SD)	3.8 (1.8)	5 (2.9)	4.5 (2.6)	**0.006**
Presence of intraplaque hemorrhage; *n* (%)	7 (13.0)	32 (41.6)	39 (29.8)	**0.001**
Presence of plaque ulceration; *n* (%)	9 (16.7)	26 (33.8)	35 (26.7)	**0.048**
Plaque SUVmax (g/ml); mean (SD)	2.2 (1.4)	2.1 (1.4)	2.1 (1.4)	0.777
SCAIL score; median (IQR)	2 (2, 2)	3 (2, 4)	3 (2, 3)	**<0.001**
90-day stroke; *n* (%)	4 (7.4)	10 (13.0)	14 (10.7)	**0.032**
90-day TIA or MACE; *n* (%)	17 (31.5)	24 (31.2)	41 (31.3)	1.000

SD, standard deviation; SCAIL, symptomatic carotid atheroma inflammation lumen-stenosis; SUVmax, maximum standardized uptake value; TIA, transient ischemic attack; MACE, major adverse cardiovascular events. Bold values indicate statistical significance.

The mean (SD) age of the study population was 64.9 (11.5) years, and 111 (84.7%) patients were male. Most patients were Chinese (91, 69.5%). The qualifying events in the study were TIA (39 patients, 29.8%) and acute ischemic stroke (92 patients, 70.2%). A total of 77 (58.8%) patients had 70%−99% stenosis in the symptomatic carotid artery. The mean (SD) symptomatic plaque SUVmax was 2.1 (1.4) g/ml and the median (IQR) symptomatic plaque SCAIL score was 3 (2, 3).

The commonest cardiovascular risk factor was hypertension (95 patients, 72.5%), followed by diabetes mellitus (77 patients, 58.8%) and being a current smoker (64 patients, 48.9%). The median (IQR) duration between the index cerebral ischemic event and recruitment to the study was 1 (0, 2.5) days. The median (IQR) duration between the index cerebral ischemic event and FDG-PET was 5 (4, 7) days. A total of 44 (33.6%) patients underwent carotid revascularization during the study period (carotid endarterectomy 32 patients, stenting 12 patients). The median (IQR) duration between the qualifying event and carotid revascularization was 11.5 (5, 28.25) days.

### Severity of stenosis, plaque FDG uptake, SCAIL, and their association with recurrent ischemic events

A total of 14 (10.7%) patients reached the primary outcome of 90-day ipsilateral ischemic stroke, with the median (IQR) duration between the qualifying event and stroke being 18.5 (11, 26) days. On the other hand, 41 (31.3%) patients reached the secondary outcome of 90-day TIA or MACE (TIA 10 patients, stroke 21 patients, acute myocardial infarction 14 patients, cardiovascular mortality 0 patients), with the median (IQR) duration between the qualifying event and the first TIA or MACE being 21 (11.5, 39) days. The two postoperative strokes which occurred during the study period but shortly after carotid revascularization were not counted toward the primary and secondary outcomes.

On multivariate time-to-event analysis adjusting for potential confounders, stenosis severity, SCAIL score, and SUVmax were all significantly associated with 90-day ipsilateral ischemic stroke (Table 2). However, for 90-day TIA or MACE, there was a statistically significant association with the SCAIL score and SUVmax, but not with stenosis severity (Table 3). On subgroup analysis of patients with moderate (50%−69%) stenosis only, the SCAIL score and SUVmax were significantly associated with both 90-day ipsilateral ischemic stroke and TIA or MACE (Supplementary Table 1).

**Table 2:** Cox proportional-hazards models analyzing the association between 90-day ipsilateral ischemic stroke and symptomatic plaque stenosis severity, SCAIL score, and SUVmax.

**Exposure**	**Model I**		**Model I** + **plaque size**		**Model I** + **presence of intraplaque hemorrhage**		**Model I** + **presence of plaque ulceration**		**Model I** + **HS-CRP level**	
	**HR (95% CI)**	***p*** **-Value**	**HR (95% CI)**	***p*** **-Value**	**HR (95% CI)**	***p*** **-Value**	**HR (95% CI)**	***p*** **-Value**	**HR (95% CI)**	***p*** **-Value**
Symptomatic artery 70%−99% stenosis	3.68 (1.14, 11.9)	**0.029**	4.18 (1.22, 14.3)	**0.023**	3.55 (1.06, 11.9)	**0.040**	3.62 (1.10, 11.9)	**0.034**	4.09 (1.20, 14.0)	**0.025**
Symptomatic plaque SCAIL score	2.28 (1.36, 3.83)	**0.002**	2.28 (1.36, 3.83)	**0.002**	2.76 (1.46, 5.22)	**0.002**	2.24 (1.34, 3.76)	**0.002**	2.29 (1.36, 3.88)	**0.002**
Symptomatic plaque SUVmax	5.92 (2.76, 12.7)	**<0.001**	6.02 (2.77, 13.1)	**<0.001**	6.63 (3.01, 14.6)	**<0.001**	6.13 (2.77, 13.6)	**<0.001**	6.08 (2.79, 13.2)	**<0.001**

Model I: Adjusted for age, sex, hypertension, diabetes mellitus, and a history of smoking.

SCAIL, symptomatic carotid atheroma inflammation lumen-stenosis; SUVmax, maximum standardized uptake value; HS-CRP, High-sensitivity C-reactive protein (mg); HR, hazard ratio; CI, confidence interval; TIA, transient ischemic attack; MACE, major adverse cardiovascular event. Bold values indicate statistical significance.

**Table 3:** Cox proportional-hazards models analyzing the association between 90-day TIA or MACE and symptomatic plaque stenosis severity, SCAIL score, and SUVmax.

**Exposure**	**Model I**		**Model I** + **plaque size**		**Model I** + **presence of intraplaque hemorrhage**		**Model I** + **presence of plaque ulceration**		**Model I** + **HS-CRP level**	
	**HR (95% CI)**	***p*** **-Value**	**HR (95% CI)**	***p*** **-Value**	**HR (95% CI)**	***p*** **-Value**	**HR (95% CI)**	***p*** **-Value**	**HR (95% CI)**	***p*** **-Value**
Symptomatic artery 70%−99% stenosis	0.94 (0.50, 1.76)	0.842	0.90 (0.47, 1.70)	0.736	0.98 (0.51, 1.88)	0.955	0.89 (0.47, 1.67)	0.710	0.92 (0.49, 1.73)	0.794
Symptomatic plaque SCAIL score	2.81 (2.02, 3.90)	**<0.001**	2.82 (2.04, 3.90)	**<0.001**	3.00 (2.07, 4.34)	**<0.001**	2.78 (2.01, 3.85)	**<0.001**	2.80 (2.02, 3.88)	**<0.001**
Symptomatic plaque SUVmax	3.33 (2.27, 4.90)	**<0.001**	3.33 (2.27, 4.90)	**<0.001**	3.29 (2.23, 4.85)	**<0.001**	3.28 (2.23, 4.82)	**<0.001**	3.33 (2.26, 4.89)	**<0.001**

Model I: Adjusted for age, sex, hypertension, diabetes mellitus, and a history of smoking.

SCAIL, symptomatic carotid atheroma inflammation lumen-stenosis; SUVmax, maximum standardized uptake value; HS-CRP, High-sensitivity C-reactive protein (mg); HR, hazard ratio; CI, confidence interval; TIA, transient ischemic attack; MACE, major adverse cardiovascular event. Bold values indicate statistical significance.

On comparison of the predictive performances of the SCAIL score and SUVmax, SUVmax was found to be superior to the SCAIL score for predicting both 90-day ipsilateral ischemic stroke (AUC: SCAIL = 0.79, SUVmax = 0.92; *p* < 0.001; 95% CI = 0.072, 0.229) and 90-day TIA or MACE (AUC: SCAIL = 0.76, SUVmax = 0.84; *p* = 0.009; 95% CI = 0.020, 0.143).

For the prediction of 90-day ipsilateral ischemic stroke, the threshold SCAIL score that gave the optimal balance of sensitivity (93.8%) and specificity (53.9%) was 3, while the threshold SUVmax that gave the optimal balance of sensitivity (93.8%) and specificity (79.1%) was 2.88 g/ml.

For the prediction of 90-day TIA or MACE, the threshold SCAIL score that gave the optimum balance of sensitivity (78.0%) and specificity (60.0%) was 3, while the threshold SUVmax that gave the optimum balance of sensitivity (73.2%) and specificity (88.9%) was 2.84 g/ml.

Further supplementary analyses found that HS-CRP level (a marker of systemic inflammation) was not predictive of symptomatic plaque SUVmax (*p* = 0.925) and both 90-day ipsilateral ischemic stroke (*p* = 0.534) and TIA or MACE (*p* = 0.818). Symptomatic plaque SUVmax was also not significantly associated with plaque characteristics (stenosis severity, plaque size, intraplaque hemorrhage, plaque ulceration) on univariate linear regression.

## Discussion

In this study, we found that stenosis severity, the SCAIL score, and SUVmax were all independently associated with 90-day ipsilateral ischemic stroke and/or TIA/MACE, validating the findings of prior studies conducted in Caucasian patients (2, 5). However, SUVmax was found to be superior to the SCAIL score in predicting 90-day events.

We also found that the optimal threshold symptomatic plaque SUVmax and SCAIL score for the prediction of 90-day ipsilateral ischemic stroke were 2.88 g/ml and 3 respectively. These thresholds are similar to the findings of prior studies, which reported optimal threshold values of 2.84 g/ml and 3 respectively for the prediction of 90-day ipsilateral ischemic stroke (2, 5).

As carotid plaque inflammation is associated with recurrent stroke, therapies to address the plaque inflammation could reduce the risk of recurrent stroke. Possible interventions include anti-inflammatory agents such as colchicine (15–17), and surgical interventions such as carotid endarterectomy and carotid stenting. Although logistically challenging, future studies should evaluate whether plaque SUVmax and SCAIL score improve patient selection for anti-inflammatory therapy and carotid revascularization.

Certain limitations of our study merit mention. First, while FDG-PET appears to be a useful clinical adjunct, the high cost of FDG-PET and limited availability may affect the generalized applicability of our findings (18). Other modalities to quantify plaque inflammation such as magnetic resonance imaging with contrast may offer a cheaper alternative, but data regarding its use is limited (19). Systemic markers of inflammation may *a priori* be a potential proxy biomarker for plaque inflammation and therefore recurrent stroke. However, evidence regarding the association between systemic biomarkers of inflammation and recurrent stroke has been conflicting (20–25). Crucially, in our study, there was no statistically significant association between baseline HS-CRP value and 90-day recurrent cerebral ischemic events. However, this could be related to the significant duration elapsed between the index clinical event and measurement of HS-CRP level (mean 6.4 days). Second, patients who underwent early carotid revascularization during the early period after the index ischemic event were not included in our analysis. It is well known that the risk of recurrent cerebral ischemia is greatest within the first few days of the initial event, and therefore early administration of optimal medical and interventional measures is important. Since carotid revascularization is performed in an expedited manner, many patients could not be included in the study. Nevertheless, our study provides important information that reliable risk-stratification may be performed even beyond the first few days after the index ischemic event to select the high-risk patients (especially when the stenosis is <70%) for various revascularization therapies.

## Conclusion

Plaque inflammation as quantified on FDG-PET may serve as a reliable biomarker for risk stratification among patients with ECAD and recent TIA or ischemic stroke. Future studies should evaluate whether patients with significant plaque inflammation as quantified on FDG-PET benefit from carotid revascularization and/or anti-inflammatory therapy.

## Data availability statement

The raw data supporting the conclusions of this article will be made available by the authors, without undue reservation.

## Ethics statement

The studies involving human participants were reviewed and approved by Domain-Specific Review Board, National Healthcare Group. The patients/participants provided their written informed consent to participate in this study.

## Author contributions

All authors listed have made a substantial, direct, and intellectual contribution to the work and approved it for publication.

## Supplementary material

Supplementary Figure 1 Flowchart illustrating the cohort selection process.

Supplementary Table 1 Univariate Cox proportional-hazards models analyzing the association between the SCAIL score and SUVmax and 90-day outcomes among patients with moderate (50%−69%) stenosis only (*n* = 54).
