# Supplementary figures and images for: Role of plaque inflammation in symptomatic carotid stenosis
Source: Front Neurol. 2023 Jan 24;14:1086465. doi: 10.3389/fneur.2023.1086465 (PMC9902904; doi:10.3389/fneur.2023.1086465)

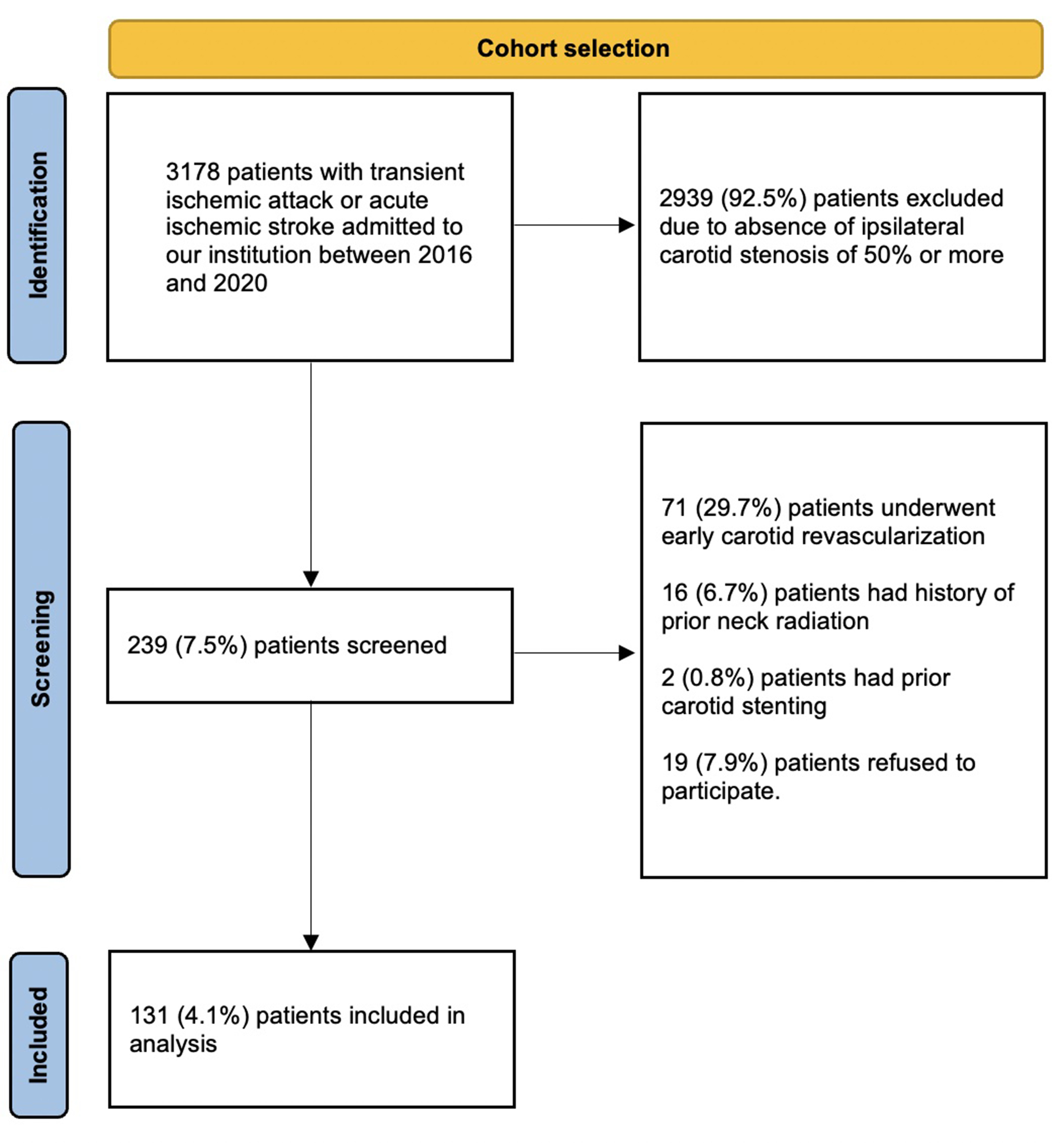

Supplement: Supplementary Figure 1 — Flowchart illustrating the cohort selection process. [file Image_1.JPEG]
