# Supplementary material for: Role of plaque inflammation in symptomatic carotid stenosis
Source: Front Neurol. 2023 Jan 24;14:1086465. doi: 10.3389/fneur.2023.1086465 (PMC9902904; doi:10.3389/fneur.2023.1086465)
Supplement: Supplementary Table 1 — Univariate Cox proportional-hazards models analyzing the association between the SCAIL score and SUVmax and 90-day outcomes among patients with moderate (50%−69%) stenosis only (n = 54). [file Table_1.docx]

Supplementary Table 1: Univariate Cox proportional-hazards models analyzing the association between the SCAIL score and SUVmax and 90-day outcomes among patients with moderate (50-69%) stenosis only (n=54)

| Exposure^*^ | **90-day ipsilateral ischemic stroke** | | **90-day TIA or MACE** | |
| --- | --- | --- | --- | --- |
|  | **HR (95% CI)** | **p-value** | **HR (95% CI)** | **p-value** |
| Symptomatic plaque SCAIL score | 3.29 (1.13, 9.53) | **0.028** | 2.70 (1.72, 4.24) | **<0.001** |
| Symptomatic plaque SUVmax | 3.91 (1.04, 14.6) | **0.043** | 2.20 (1.44, 3.64) | **<0.001** |

^*^Multivariate analysis was not performed due to concerns over overfitting, in view of the small sample size (n=54).

Abbreviations: SCAIL, symptomatic carotid atheroma inflammation lumen-stenosis; SUVmax, maximum standardized uptake value; TIA, transient ischemic attack; MACE, major adverse cardiovascular event; HR, hazard ratio; CI, confidence interval.
